# Reassessing the pathogenicity of c.2858G>T(p.(G953V)) in *COL4A5* Gene: report of 19 Chinese families

**DOI:** 10.1038/s41431-019-0523-1

**Published:** 2019-10-01

**Authors:** Yanqin Zhang, Jie Ding, Suxia Wang, Hongwen Zhang, Xuhui Zhong, Xiaoyu Liu, Ke Xu, Fang Wang

**Affiliations:** 1 Department of Pediatrics, Peking University First Hospital, 100034 Beijing, China; 2 Department of Electron Microscopy, Peking University First Hospital, 100034 Beijing, China

**Keywords:** Genetic counselling, Disease genetics

## Abstract

X-linked Alport syndrome (XLAS) is an inherited renal disease caused by mutations in *COL4A5* gene. The c.2858G>T(p.(G953V)) in *COL4A5* gene (rs78972735) has been considered pathogenic previously. However, there are conflicting interpretations of its pathogenicity recently. Here we presented 19 Chinese families, out of which 36 individuals (18 probands and 18 family members) carried the c.2858G>T(p.(G953V)) in *COL4A5* gene. The clinical manifestations and genetic findings of them were analyzed. We found there were no clinical features of Alport syndrome not only in six probands with c.2858G>T(p.(G953V)) in *COL4A5* plus pathogenic variants in other genes (e.g., *WT1*, *ADCK4*, *NPHP1*, *TRPC6*, *COL4A4,* and *PAX2*) but also in another six probands with only the c.2858G>T(p.(G953V)) variant. The other six probands with a combination of c.2858G>T(p.(G953V)) and another pathogenic variant in *COL4A5* had XLAS. Eleven family members (11/18, nine females and two males) who had only the c.2858G>T(p.(G953V)) variant were asymptomatic. These two males (at age of 42 and 35 years) had normal result of urine analysis and no more clinical traits of Alport syndrome. We conclude c.2858G>T(p.(G953V)) in *COL4A5* gene is not a pathogenic variant for XLAS. Individuals should not be diagnosed as XLAS only based on the detection of c.2858G>T(p.(G953V)) in *COL4A5* gene.

## Introduction

Alport syndrome (AS) is an inherited renal disease characterized by hematuria, proteinuria, and progressive renal failure, variably associated with hearing loss and ocular abnormalities [1–3]. It is caused by mutations in the *COL4A3*, *COL4A4*, or *COL4A5* genes encoding α3, α4, and α5 chains of collagen type IV, a component of the glomerular basement membrane (GBM) in the kidney [4, 5]. Eighty-five percent of families have X-linked Alport syndrome (XLAS) with mutations in *COL4A5* gene [6, 7]. It is well known that male patients with XLAS, who are hemizygous for mutations in *COL4A5* gene, are severely affected [8, 9]. All of the males have microscopic hematuria and 90% of them progress to end-stage renal disease (ESRD) before age 40 years [9–12].

Up to now, 1000 mutations in *COL4A5* gene have been included in the Human Gene Mutation Database (HGMD) and about 36% of them are glycine substitutions. Glycine substitutions in the collagenous domain of the α5 (IV) chain are usually considered pathogenic, since glycine is the smallest amino acid and it is critical for the formation of the triple helical structure [13, 14]. However, many variants have quite limited evidence for pathogenicity assessment. With the wide application of next generation sequencing (NGS), more and more variations in *COL4A5* gene are found and the relationship of genotype and phenotype are analyzed [15, 16]. Therefore, the assessments of pathogenicity are important and would be corrected over time.

The c.2858G>T(p.(G953V)) in *COL4A5* gene (rs78972735) is considered pathogenic in the HGMD database, while it is considered conflicting of pathogenicity in the ClinVar database [17–20]. In addition, it is more common in Asian population, in whom the allele frequency ranges from 0.01 to 0.03. In this study, we report 19 families from China with the variant of c.2858G>T(p.(G953V)) in *COL4A5* gene and their clinical manifestations, providing more evidence to assess its pathogenicity.

## Subjects and methods

### Patients and families

We selected patients from the online registry database of hereditary kidney diseases in children in China (https://www.nrdrs.org.cn/app/rare/), according to the following two criteria (1) cases with gene mutations tested by targeted NGS; (2) cases with the variant of c.2858G>T(p.(G953V)) in *COL4A5* gene. Cases without clinical data were excluded. And all their family members in the registry database were included. The clinical data including gender, age, renal and extrarenal manifestations, and renal histopathology results were collected. The Ethical Committee of Peking University First Hospital approved the project, and informed consent was obtained from the probands and their family members.

### Targeted NGS and data analysis

Genomic DNA was isolated from the blood samples using a FlexiGene DNA Kit (Qiagen) according to the manufacturer’s protocol. Genomic DNA from probands was detected for pathogenic variants with targeted NGS (No.DX0595, including 464 genes related to inherited kidney disease) by BGI in China. Data were filtered with the variant databases, such as dbSNP (https://www.ncbi.nlm.nih.gov/projects/SNP/), the 1000 Genomes Project (ftp://ftp-trace.ncbi.nih.gov/1000genomes/ftp/release), and Exome Aggregation Consortium (ExAC) (http://exac.broadinstitute.org/). The allele frequency of the candidate variants were considered to be <5% in these databases. The functional significance of unpublished variants was predicted using SIFT, PolyPhen 2, and Mutation Taster. The evolutionary conservation of the variant sites was evaluated using the PhyloP Primates tool. Splice site effect prediction was performed using Human Splicing Finder (Version 3.1) [21]. The pathogenicity of variants identified was classified according to the ACMG standards and guidelines [22]. If the following criteria were met including truncating mutations (nonsense, consensus splice site ± 1 or 2 nucleotide, large deletion, and frameshift) or novel missense variants in domain of high evolutionary conservation and more than two prediction scores classified the allele as disease causing (SIFT, Mutation Taster, Polyphen 2), the variant was ascertained to be a pathogenic variant. All the variants data has been submitted to ClinVar (Submission ID: SUB6246401; Organization ID: 505424).

Sanger sequencing or real-time quantitative PCR (qPCR) was used to confirm candidate pathogenic variants identified with NGS in probands. Genomic DNA from family members was analyzed by Sanger sequencing or qPCR to determine whether they had the same pathogenic variants as the probands.

## Results

Nineteen different Chinese families (51 individuals) were enrolled in this study, out of which 36 individuals carried the c.2858G>T(p.(G953V)) in *COL4A5* gene. The clinical features and genetic results of the individuals enrolled in this study were shown in Table 1.

**Table 1:** Clinical characteristics and nucleotide variants found in the probands and their family members

Family number	Family member	Sex	Age, years	Kidney disease (age, years^a^)	Hearing loss (age, years^a^)	Ocular lesions	Renal Pathology	Variants (nucleotide change; effect on protein)		
								*COL4A5*:c.2858G>T;p.(Gly953Val)	Other variants	
1	Proband	F	10	PU (8), ESRD (10)	No	No	FSGS	+	*ADCK4*:c.448C>T; p. (Arg150*)	*ADCK4*:c.748G>C;p.(Asp250His)
1	Mother	F	31	None	No	No	None	NT	+	−
1	Father	M	33	None	No	No	None	NT	−	+
1	Sister	F	Dead, 7, ESRD	ESRD (<7)	NA	NA	FSGS	NT	NT	NT
2	Proband	M	1 month	CNS	NA	NA	None	NT	NT	NT
2	Mother	F	25	None	No	No	None	+	*NPHS1*:c.3027C>G;p.(Tyr1009*)	−
2	Father	M	27	None	No	No	None	NT	−	*NPHS1*:c.144dup;p.(Val49Serfs*43)
3	Proband	F	9	PU (9), CRF (9)	No	No	FSGS	+	*TRPC6*:c.335C>T; p.(Pro112Leu)	
4	Proband	M	2	PU (2), CRF (2), Hypospadia	No	No	MsPGN	+	*WT1*:c.1432+5G>A	
4	Mother	F	24	None	No	No	None	+	−	
4	Father	M	25	None	No	No	None	−	−	
5	Proband	F	16	PU (10), ESRD (16)	No	Microphthalmus	None	+	*PAX2*:c.418C>T;p.(Arg140Trp)	
5	Mother	F	44	None	No	No	None	+	−	
5	Father	M	42	None	No	No	None	−	−	
6	Proband	F	13	CRF (13)	No	No	MsPGN, FSGS	+	*NPHP1*:c.-94_*455del	
6	Mother	F	42	None	No	No	None	−	+	
6	Father	M	42	None	No	No	None	+	−	
7	Proband	F	3	HU (3)	No	No	None	+	*COL4A4*:c.4129C>T;p.(Arg1377*)	
7	Mother	F	33	HU (29)	No	No	None	+	+	
7	Father	M	34	None	No	No	None	−	−	
8	Proband	M	2	HU (2), PU (2)	No	No	MsPGN	+	*COL4A5*:c.834+5G>A	
8	Mother	F	24	HU, PU	No	No	None	+	+	
9	Proband	F	6	HU (4), PU (4)	No	No	AS	+	*COL4A5*:c.937-2A>C	
9	Mother	F	45	None	No	No	None	+	−	
9	Father	M	46	None	No	No	None	−	−	
9	Sister	F	20	None	No	No	None	+	−	
10	Proband	M	5	HU (3), PU (5)	No	No	AS	+	*COL4A5*:c.1813_1814dup; p.(Gly606Leufs*13)	
10	Mother	F	32	HU	No	No	None	+	+	
11	Proband	M	5 months	HU, PU	No	No	None	+	*COL4A5*:c.4024G>T; p.(Gly1342*)	
11	Mother	F	39	HU, PU	No	No	None	+	+	
11	Father	M	41	None	No	No	None	−	−	
11	Sister	F	15	HU (2), PU (2)	No	No	None	NT	NT	
12	Proband	M	9	HU (5), PU (5)	No	No	AS	+	*COL4A5*:c.4510delG; p.(Ala1504Profs*50)	
12	Mother	F	44	HU	No	No	None	+	+	
12	Father	M	45	None	No	No	None	−	−	
13	Proband	M	14	HU (4), PU (14)	Yes(14)	No	AS	+	*COL4A5*:c.81G>A; p.(Ala27=)	
13	Mother	F	40	HU, PU	No	No	None	+	+	
14	Proband	M	13	NS (2), steroid sensitive	No	No	None	+		
14	Mother	F	43	None	No	No	None	+		
15	Proband	M	2	NS (1.5), steroid resistance	No	No	MsPGN	+		
15	Mother	F	40	None	No	No	None	+		
16	Proband	F	11	ESRD (9)	No	No	None	+		
16	Mother	F	47	None	No	No	None	+		
17	Proband	M	6	ESRD (5)	No	No	None	+		
17	Mother	F	30	None	No	No	None	+		
18	Proband	M	5	NS (4), steroid resistance	No	Cataract due to steroid therapy	MsPGN	+		
18	Mother	F	36	None	No	No	None	+		
18	Father	M	33	None	No	No	None	−		
19	Proband	F	6	NS (6), steroid resistance	No	No	FSGS	+		
19	Mother	F	35	None	No	No	None	−		
19	Father	M	35	None	No	No	None	+		

+ Variant present; − Variant not present; the reference sequences: ADCK4 (NG_027800.1, NM_024876.3); NPHS1 (NG_013356.2, NM_004646.3); TRPC6 (NG_011476.2, NM_004621.5); WT1 (NG_009272.1, NM_024426.4); PAX2 (NG_008680.2, NM_003990.3); NPHP1 (NG_008287.1, NM_000272.3); COL4A4 (NG_011592.1, NM_000092.4); COL4A5 (NG_011977.2, NM_033380.2)

*F* female, *M* male, *CNS* congenital nephrotic syndrome, *HU* hematuria, *PU* proteinuria, *NS* nephrotic syndrome, *ESRD* end-stage renal disease, *CRF* chronic renal failure, *FSGS* focal segmental glomerulosclerosis, *MsPGN* mesangial proliferative glomerulonephritis, *AS* Alport syndrome, *NA* data missing, *NT* gene not tested

^a^Age at diagnosis

### The 19 families were divided into three groups according to their genetic findings

#### Group 1: families with the c.2858G>T(p.(G953V)) variant plus pathogenic variants in other genes

There were seven families in group 1 (family number 1–7). The proband from family 1, a 10-year-old girl with ESRD, had a younger sister who died at age of 7 years due to ESRD. Both of the two sisters had focal segmental glomerulosclerosis (FSGS). Genetic test of the proband revealed compound heterozygous variants in *ADCK4* gene and the c.2858G>T(p.(G953V)) variant. And her parents were asymptomatic heterozygous carriers of the variants in *ADCK4*. The probands from two families (family number 3 and 6), both of them were female, presented with renal failure at an early age, identified with pathogenic variants in *TRPC6* gene and *NPHP1* gene respectively, in addition to the c.2858G>T(p.(G953V)) variant. Another two probands (family number 4 and 5) presented with hypospadia and microphthalmus, respectively. Genetic test found de novo pathogenic variants in *WT* gene and *PAX2* gene in these two probands, respectively, besides the c.2858G>T(p.(G953V)) variant inherited from their mothers. The proband from family 7 presented with hematuria at age of 3 years, with a positive family history of hematuria in her mother. Genetic test found a heterozygous nonsense variant in *COL4A4* gene and the c.2858G>T(p.(G953V)) variant in both the proband and her mother. The last proband (family number 2) in group 1 was diagnosed with congenital nephrotic syndrome (CNS) at age of 1 month after birth. The mother and father undertook genetic test because the DNA sample was not available for proband 2. Genetic test found the parents were heterozygous carriers of the pathogenic variants in *NPHS1*. Moreover, the mother had the c.2858G>T(p.(G953V)) variant but she was asymptomatic.

#### Group 2: families with the c.2858G>T(p.(G953V)) variant plus another pathogenic variant in COL4A5 gene

There were six families in group 2 (family number 8–13). All of the six probands in this group presented with hematuria and proteinuria. One of the proband from family 13 presented with hearing loss at age of 14 years. Five of the probands underwent renal biopsy, and four had typical GBM changes of AS. Five of the mothers had hematuria with or without proteinuria, except the mother from family 9. Genetic test found a pathogenic variant in *COL4A5* gene and the c.2858G>T(p.(G953V)) variant in each of the six probands. Both of the two variants in *COL4A5* gene in five probands (except proband 9) were inherited from their mothers. It was proved the pathogenic variant c.937-2A>C in proband 9 was de novo, and the c.2858G>T(p.(G953V)) variant was inherited from her mother.

#### Group 3: families with only the c.2858G>T(p.(G953V)) variant

There were six families in group 3 (family number 14–19). Three of the probands in this group presented with steroid resistant nephrotic syndrome. The onset age of nephrotic syndrome in these three probands was 1.5, 4, and 6 years, respectively. The result of kidney biopsy in these three probands was FSGS and mesangial proliferative glomerulonephritis (MsPGN) without anomalies of GBM. Two of the probands presented with ESRD at age of 9 and 5 years, respectively. The proband from family 14 presented with steroid sensitive nephrotic syndrome at age of 2 years. All these six probands had no hematuria and no family history of kidney diseases. Genetic test found the c.2858G>T(p.(G953V)) variant in *COL4A5* gene in all these six probands. The c.2858G>T(p.(G953V)) variant was inherited from their mothers in five of the probands except proband 19, who inherited this variant from her healthy father.

### Clinical features of the family members with the c.2858G>T(p.(G953V)) variant

There were 18 family members identified with the c.2858G>T(p.(G953V)) variant in this study. They were 2 males and 16 females. In the male group, they were two fathers (family number 6 and 19) with normal result of urine analysis at age of 42 and 35 years, respectively. And both of the two fathers had no extrarenal manifestations. In the female group, in addition to the heterozygous c.2858G>T(p.(G953V)) variant, a heterozygous pathogenic variant of *COL4A5* gene was found in five of them (5/16) and a heterozygous pathogenic variant of *COL4A4* gene was found in one of them (1/16). These six females presented with hematuria with or without proteinuria at age of 24–44 years and with normal renal function. Another female identified with a heterozygous p.(Tyr1009*) variant in *NPHS1* gene and the heterozygous c.2858G>T(p.(G953V)) variant in *COL4A5* was asymptomatic. The other nine females who had the heterozygous c.2858G>T(p.(G953V)) variant were all asymptomatic at age of 20–47 years. And the clinical features of the individuals with only c.2858G>T(p.(G953V)) variant in *COL4A5* gene were shown separately in Table 2.

**Table 2:** Clinical characteristics of the individuals with only c.2858G>T(p.(G953V)) variant in *COL4A5* gene

Family number	Family member	Sex	Age, years	Hematuria	Proteinuria (age, years^a^)	ESRD (age, years^a^)	Hearing loss (age, years^a^)	Ocular lesions	Renal pathology
6	Father	M	42	No	No	No	No	No	None
14	Proband	M	13	No	NS (2), steroid sensitive	No	No	No	None
15	Proband	M	2	No	NS (1.5), steroid resistance	No	No	No	MsPGN
17	Proband	M	6	No	No	ESRD (5)	No	No	None
18	Proband	M	5	No	NS (4), steroid resistance	No	No	Cataract due to steroid therapy	MsPGN
19	Father	M	35	No	No	No	No	No	None
4	Mother	F	24	No	No	No	No	No	None
5	Mother	F	44	No	No	No	No	No	None
9	Mother	F	45	No	No	No	No	No	None
9	Sister	F	20	No	No	No	No	No	None
14	Mother	F	43	No	No	No	No	No	None
15	Mother	F	40	No	No	No	No	No	None
16	Proband	F	11	No	No	ESRD (9)	No	No	None
16	Mother	F	47	No	No	No	No	No	None
17	Mother	F	30	No	No	No	No	No	None
18	Mother	F	36	No	No	No	No	No	None
19	Proband	F	6	No	NS (6), steroid resistance	No	No	No	FSGS

*F* female, *M* male, *NS* nephrotic syndrome, *ESRD* end-stage renal disease, *FSGS* focal segmental glomerulosclerosis, *MsPGN* mesangial proliferative glomerulonephritis

^a^Age at diagnosis

### Gene analysis

Fifteen pathogenic variants were detected in the 19 families in this study besides the c.2858G>T(p.(G953V)) variant (Table 3). Seven of them (7/15) were novel pathogenic variants: one in *NPHS1* gene c.144dup (p.(Val49Serfs*43)), one in *TRPC6* gene c.335C>T (p.(Pro112Leu)), one in *PAX2* gene c.418C>T (p.(Arg140Trp)), and four in *COL4A5* gene c.937-2A>C, c.1813_1814dup (p.(Gly606Leufs*13)), c.4024G>T (p.(Gly1342*)), and c.4510delG (p.(Ala1504Profs*50)). The variant c.81G>A (p.(Ala27=)) in *COL4A5* was not found in 1000G and ExAC. The mutation Taster predicted it as disease causing. The c.81G>A occurs in the last base pair of exon 1 of *COL4A5*. Human Splicing Finder predicted it as broken WT donor site and most probably affecting splicing. The c.2858G>T(p.(G953V)) variant (rs78972735) in *COL4A5* gene had a high allele frequency of 0.01 in the 1000G Asian populations. It was included in the ClinVar, HGMD, and LOVD database. The mutation Taster predicted the c.2858G>T(p.(G953V)) variant as polymorphism, while Polyphen 2 and SIFT scored it as damaging.

**Table 3:** Molecular features and predicted pathogenicity of DNA variants found in this study

DNA variant	Effect on protein	Type	dbSNP reference ID	ClinVar	HGMD	LOVD	1000 G ASN AF	ExAC AF	SIFT (score)	PolyPhen 2 (score)	Mutation taster	Human splicing finder
*ADCK4*												
c.448C>T	p.(Arg150*)	Nonsense	–	–	–	–	–	–	–	–	DC	–
c.748G>C	p.(Asp250His)	Missense	–	–	–	–	–	–	D (0)	PD (1)	DC	–
*NPHS1*												
c.3027C>G	p.(Tyr1009*)	Nonsense	rs762184939	P	DM	–	–	–	–	–	DC	–
c.144dup	p.(Val49Serfs*43)	Frameshift	–	–	–	–	–	–	–	–	DC	–
*TRPC6*												
c.335C>T	p.(Pro112Leu)	Missense	–	–	a	–	–	–	D (0)	PD (1)	DC	–
*WT1*												
c.1432+5G>A	–	Splicing	–	–	DM	–	–	–	–	–	–	SL
*PAX2*												
c.418C>T	p.(Arg140Trp)	Missense	–	–	b	–	–	–	D (0)	PD (1)	DC	–
*NPHP1*												
c.-94_^a^455del	–	Large deletion	–	P	DM		–	–	–	–	–	–
*COL4A4*												
c.4129C>T	p.(Arg1377*)	Nonsense	rs121912861	P	DM	P	–	2E−05	–	–	DC	–
*COL4A5*												
c.834+5G>A	–	Splicing	–	P	DM	LP	–	–	–	–	–	SL
c.937-2A>C	–	Splicing	–	–	–	–	–	–	–	–	–	SL
c.1813_1814dup	p.(Gly606Leufs*13)	Frameshift	–	–	–	–	–	–	–	–	DC	–
c.4024G>T	p.(Gly1342*)	Nonsense	–	–	c	–	–	–	–	–	DC	–
c.4510delG	p.(Ala1504Profs*50)	Frameshift	–	–	–	–	–	–	–	–	DC	–
c.81G>A	p.(Ala27=)	Coding-synon	–	U	–	–	–	–	–	–	DC	SL
c.2858G>T	p.(Gly953Val)	Missense	rs78972735	U^d^	DM	U	0.01	0.003	D (0)	PD (0.99)	Polymorphism	–

Pathogenicity of missense variants predicted using SIFT, MutationTaster and PolyPhen 2

*P* pathogenic, *LP* likely pathogenic, *PD* probably damaging, *D* deleterious, *DC* disease causing, *DM* disease-causing mutation, *SL* loss of canonical splice site, *U* uncertain significance

^a^p.(Pro112Gln) has been reported

^b^p.(Arg140Gln) has been reported

^c^p.(Gly1342Arg) has been reported

^d^Conflicting interpretations of pathogenicity

## Discussion

In this study, we reported 19 Chinese families with the c.2858G>T(p.(G953V)) variant in *COL4A5* gene. By analyzing the clinical manifestations of 36 individuals (18 probands and 18 family members) who carried the c.2858G>T(p.(G953V)) variant, we found the c.2858G>T(p.(G953V)) variant in *COL4A5* gene was not a pathogenic variant resulting XLAS.

The 19 families enrolled in this study were divided into three groups for analysis. We found probands in group 1 and group 3 presented mainly with early onset of renal failure (median age of 9 years) or nephrotic syndrome. The proband from family 14 in group 3 presented steroid sensitive nephrotic syndrome without hematuria and got complete remission after treatment. In addition, the available renal biopsies of the probands in these two groups were FSGS or MsPGN without abnormal changes of GBM. And the majority of the families in these two groups had no family history of hematuria and had no hearing loss or typical ocular lesions for AS [23, 24]. Apparently the probands in group 1 and group 3 should not be diagnosed as XLAS even all of them had the c.2858G>T(p.(G953V)) variant in *COL4A5* gene. Moreover, eleven (nine females and two males) of the family members who had only the c.2858G>T(p.(G953V)) variant were asymptomatic. The two males (at age of 42 and 35 years) had normal result of urine analysis and no more clinical traits of AS. This strongly suggested that the c.2858G>T(p.(G953V)) variant in *COL4A5* gene was not a pathogenic variant for XLAS. In a recently report [19] of a family with polycystic kidney disease, the c.2858G>T(p.(G953V)) variant in *COL4A5* gene was identified in four individuals who had no clinical features of AS, which supported our findings.

Totally 11 female and 6 male individuals with only c.2858G>T(p.(G953V)) variant in *COL4A5* gene were included in this study. Two of the 11 females, of the youngest ages, developed ESRD at 9-year old and steroid resistance nephrotic syndrome at 6-year old, respectively. And the other nine females aged from 20 to 47 years old had normal urinary analysis. This is different from the natural history of female patients with XLAS. It has been reported that hematuria, usually microscopic, presented in 96% of females with XLAS and proteinuria developed in 72.6–75% [8, 25]. Especially a median renal survival age of 65.0 years in females with XLAS was reported [8]. Thus, ESRD at 9-year old in female is too early to diagnose as XLAS. In the other hand, hematuria is a common clinical feature in all males with XLAS and 90% of male patients are at risk of developing ESRD before the age of 40 [9]. However, all the six males with only c.2858G>T(p.(G953V)) variant in *COL4A5* gene had no hematuria. Furthermore, two of the males had normal result of urine analysis at age of 35 and 42. Therefore, no clues to support diagnosis of XLAS in all of the 17 individuals who detected with only c.2858G>T(p.(G953V)) variant in *COL4A5* gene, especially for males. In addition, the variant c.2858G>T(p.(G953V)) in *COL4A5* gene has a high allele frequency of more than 0.01 in 1000 Genomes, gnomAD—Genomes, gnomAD—Exomes and ExAC, which implies its benign possibility.

By reviewing of patients diagnosed as XLAS and detected with c.2858G>T(p.(G953V)) in *COL4A5* gene previously reported [6, 18, 20], we found that totally four patients have been reported. In three of them, *COL4A5* gene was tested by RT-PCR and direct sequencing. And in each of the three patients, c.2858G>T(p.(G953V)) in *COL4A5* gene and another pathogenic mutation in *COL4A5* gene were detected on the same allele in the same family [6, 18]. Only in one patient, the three Alport genes were detected by NGS. And the variant c.2858G>T(p.(G953V)) in *COL4A5* gene and a splicing variant c.3817+1G>T in *COL4A4* gene were detected [20]. Together with the individuals in group 2 in this study, the clinical manifestations of AS in these patients may not be caused by c.2858G>T(p.(G953V)) in *COL4A5* gene. Further information and researches are required to explore whether the variant c.2858G>T(p.(G953V)) in *COL4A5* gene would have a modifier effect in the phenotype.

Glycine substitutions in a collagenous domain of the a (IV) chain are usually disease causing variants. However, a few of Glycine substitutions have been considered neutral polymorphisms, including c.127G>C (p.Gly43Arg) in *COL4A3*, c.2996G>A (p.Gly999Glu) in *COL4A4*, c.1634G>C (p.Gly545Ala) in *COL4A4*. Accompanied by the wide application of NGS in diagnosis of AS, we would get a better understanding of the pathogenicity of variants in Alport genes. And in order to make an accurate diagnosis, the gene results must combined with clinical manifestations.

Therefore, we should carefully use the criteria “a pathogenic mutation in the *COL4A5* gene” to confirm the diagnoses of XLAS [13, 24, 26]. Persistent glomerular hematuria should not be ignored and it is the basement for diagnoses of XLAS [9, 25, 27]. In the case with only one missense variant in *COL4A5* gene (including glycine substitutions), any of the following items including [27] positive family history of AS or renal failure, hearing loss, typical ocular lesions, typical GBM changes of AS, and abnormal staining of collagen IV α5 chain would help to avoid a misdiagnoses of XLAS.

In summary, the cases we reported here providing evidence to reassess the pathogenicity of c.2858G>T(p.(G953V)) in *COL4A5* gene. We conclude c.2858G>T(p.(G953V)) in *COL4A5* gene is not a pathogenic variant for XLAS. Individuals should not be diagnosed as XLAS only based on the detection of c.2858G>T(p.(G953V)) in *COL4A5* gene.
